# Psychosocial moderation of polygenic risk for cannabis involvement: the role of trauma exposure and frequency of religious service attendance

**DOI:** 10.1038/s41398-019-0598-z

**Published:** 2019-10-21

**Authors:** Jacquelyn L. Meyers, Jessica E. Salvatore, Fazil Aliev, Emma C. Johnson, Vivia V. McCutcheon, Jinni Su, Sally I-Chun Kuo, Dongbing Lai, Leah Wetherill, Jen C. Wang, Grace Chan, Victor Hesselbrock, Tatiana Foroud, Kathleen K. Bucholz, Howard J. Edenberg, Danielle M. Dick, Bernice Porjesz, Arpana Agrawal

**Affiliations:** 1 Henri Begleiter Neurodynamics Laboratory, Department of Psychiatry, State University of New York Downstate Medical Center, Brooklyn, NY 11203 USA; 2 Virginia Commonwealth University, Richmond, VA 232212 USA; 3 Washington University School of Medicine, St. Louis, MO 63110 USA; 4 Indiana University School of Medicine, Indianapolis, IN 46202 USA; 5 Mount Sinai School of Medicine, New York, NY 10029 USA; 6 University of Connecticut School of Medicine, Connecticut, CT 06030-2103 USA

**Keywords:** Addiction, Genomics

## Abstract

Cannabis use and disorders (CUD) are influenced by multiple genetic variants of small effect and by the psychosocial environment. However, this information has not been effectively incorporated into studies of gene–environment interaction (GxE). Polygenic risk scores (PRS) that aggregate the effects of genetic variants can aid in identifying the links between genetic risk and psychosocial factors. Using data from the Pasman et al. GWAS of cannabis use (meta-analysis of data from the International Cannabis Consortium and UK Biobank), we constructed PRS in the Collaborative Study on the Genetics of Alcoholism (COGA) participants of European (*N*: 7591) and African (*N*: 3359) ancestry. The primary analyses included only individuals of European ancestry, reflecting the ancestral composition of the discovery GWAS from which the PRS was derived. Secondary analyses included the African ancestry sample. Associations of PRS with cannabis use and DSM-5 CUD symptom count (CUDsx) and interactions with trauma exposure and frequency of religious service attendance were examined. Models were adjusted for sex, birth cohort, genotype array, and ancestry. Robustness models were adjusted for cross-term interactions. Higher PRS were associated with a greater likelihood of cannabis use and with CUDsx among participants of European ancestry (*p* < 0.05 and *p* < 0.1 thresholds, respectively). PRS only influenced cannabis use among those exposed to trauma (*R*^2^: 0.011 among the trauma exposed vs. *R*^2^: 0.002 in unexposed). PRS less consistently influenced cannabis use among those who attend religious services less frequently; PRS × religious service attendance effects were attenuated when cross-term interactions with ancestry and sex were included in the model. Polygenic liability to cannabis use was related to cannabis use and, less robustly, progression to symptoms of CUD. This study provides the first evidence of PRS × trauma for cannabis use and demonstrates that ignoring important aspects of the psychosocial environment may mask genetic influences on polygenic traits.

## Introduction

Cannabis is the most common illicit drug used in the US, and ~10–30% of cannabis users meet criteria for a cannabis use disorder (CUD) at some point in their lifetime^1,2^. Twin studies have suggested that individual differences in cannabis use and CUD are due to genetic (45–75%) and environmental factors^3^. Recent advances in gene identification via genome-wide association studies (GWASs) led by consortia and biobanks (e.g., International Cannabis Consortium (ICC)^4^, Psychiatric Genomics Consortium, UK Biobank), combined with existing theoretical frameworks for studying gene–environment interaction (GxE)^5^, permit new ways of identifying contributions of common variants to cannabis use and problems and allow for an evaluation of whether genetic susceptibility to cannabis involvement varies in the context of well-studied psychosocial risk and protective factors, such as trauma exposure and religiosity. This may aid in our understanding of genetic influences on cannabis involvement and inform translation of genomics findings into useful risk factor targets for public health and clinical intervention.

Until recently, few studies have examined genetic risk for cannabis involvement alone. Of the cannabis use and CUD studies that have been published, few robust and replicated genetic risk variants have been identified. A GWAS of Diagnostic and Statistical Manual of Mental Disorders, Fourth Edition (DSM-IV) cannabis-dependence criterion count performed in three independent substance-dependence cohorts (the Yale-Penn Study, Study of Addiction: Genetics and Environment, and International Consortium on the Genetics of Heroin Dependence^6^) implicated three independent genomic regions, including rs146091982, a variant within solute carrier *SLC35G1*, a gene which encodes a protein involved in cellular calcium levels; rs77378271, a variant within *CSMD1*, a brain expressed gene of unknown function; and in a novel antisense transcript *RP11-206M11* (rs143244591). In addition, a recent meta-analysis of GWAS on 2080 DSM-IV cannabis-dependent cases and 6435 cannabis-exposed controls identified a novel genome-wide significant region on chromosome 10 (rs1409568)^7^, which the authors show may have a role as an enhancer in addiction-relevant brain regions, such as the dorsolateral prefrontal cortex and the angular and cingulate gyri. Another recent GWAS of adults in Denmark and Iceland (iPSYCH) implicated a single-nucleotide polymorphism (SNP; rs56372821) that is a strong expression quantitative trait locus for *CHRNA2* and that *CHRNA2* expression in cerebellum is associated with CUD^8^. Gene expression profiles in the Allan Brain Atlas (http://www.brain-map.org/), which showed that, of all the genes evaluated (58,692 probes analyzed), cannabinoid receptor 1 gene (*CNR1*) demonstrated the strongest negative correlation with *CHRNA2* expression, led the authors to suggest the possibility of a biological interaction between the endocannabinoid system and alpha-2 subunit containing nicotinic acetylcholine receptor. Notably, there was no evidence of association of these previously identified cannabis risk variants with CUD in the iPSYCH study^8^. This might be due to different phenotype definitions among the studies: the Yale-Penn study^6^ analyzed association with cannabis criterion counts, and the cannabis-dependence meta-analysis^7^ used cannabis-exposed individuals as controls in their study. In addition, the composition of the cohorts analyzed also differs: the previous GWASs^6,7^ were based on cohorts established to study genetics of substance use disorders while the iPSYCH cohort^8^ is ascertained for major mental illnesses.

Larger GWAS sample sizes can be obtained by examining phenotypes that are less stringently defined and therefore more likely available across genomic studies of other outcomes. For example, the ICC published a meta-analysis of genome-wide association data of 13 cohorts (*N* = 32,330) and four replication samples (*N* = 5627) of European ancestry (EA) assessed for cannabis use (i.e., having ever used cannabis in one’s lifetime)^4^. The ICC meta-analysis included data from previous GWAS of cannabis dependence^6^ described above. While no individual SNP reached genome-wide significance, gene-based tests identified four genes significantly associated with lifetime cannabis use: *NCAM1* and *CADM2* (genes involved in neural cell adhesion), short coiled-coil protein (*SCOC*), and a potassium sodium-activated channel gene (*KCNT2*). Furthermore, they demonstrated that all common SNPs explained 13–20% (*p* < 0.001) of the variance in lifetime cannabis use. While several large studies and consortia efforts to examine cannabis involvement are underway, this is currently the largest peer-reviewed meta-analysis of cannabis GWAS studies to date. A more recent and larger meta-analysis (*N*: 184,765), including data from the ICC (*N*: 35,297), 23andme (*N*: 22,683), and the UK Biobank (*N*: 126,785), is now available from Pasman et al.^9^. The Pasman et al. study^9^ implicated *CADM2 and NCAM1* and additional genetic risk loci for cannabis ever use, including *ZNF704* (*zinc finger protein 704*), *RABEP2* (Rabaptin, RAB GTPase Binding Effector Protein 2)/*ATP2A1* (ATPase Sarcoplasmic/Endoplasmic Reticulum Ca2+ Transporting 1), and *ALDH2* (Aldehyde Dehydrogenase 2 Family Member). While some genes mentioned above have been previously identified via genetic association studies with addiction related phenotypes (e.g., ALDH2 and alcohol use disorders (AUDs)^10^), the biological mechanisms linking these genetic variants to cannabis use/abuse are largely unknown. Evidence that there are both shared and non-shared genetic contributions to substance use and substance use disorders^11^ necessitates an examination of whether individual loci and polygenic risk for cannabis ever use predicts progression to cannabis use and problems (e.g., DSM-5 disorder criteria) in independent samples. In addition, evidence that genetic influences on other substance use and disorders are modified by the social environment^12,13^ suggests that key psychosocial factors may aid in our understanding of genetic influences on cannabis involvement.

An early GxE study found that greater religiosity diminished latent genetic influences on alcohol use initiation^14^. Since then, genetically informed resilience research has identified several aspects of religiosity as important protective factors against substance use behaviors^15–17^, which may limit the expression of genetic propensity for CUD. This can be interpreted in the context of Shanahan and Hofer’s *social context as social control*^5^, which refers to protective environments that may attenuate a genetic predisposition toward a negative outcome. In contrast, *contextual triggering*^5^ was used to describe a detrimental environment combining with a genetic predisposition toward a negative outcome. For example, adverse experiences have been shown to exacerbate genetic influences on substance use and disorders^18–20^, including CUD^17,21,22^. We note that even the association of Alcohol Dehydrogenase 1B (Class I), Beta Polypeptide (*ADH1B*)-rs1229984 with alcohol use/problems, and Cholinergic Receptor Nicotinic Alpha 5 Subunit (*CHRNA5*)-rs16969968 with cigarette smoking, two of the most robust and replicable genetic effects in the substance use literature, have been found to differ as a function of trauma exposure^13,23,24^. However, critiques of early GxE studies pointed to the likelihood of false positive findings given the focus on candidate genes and small sample sizes typical of many well-characterized (i.e., deeply phenotyped) genetic studies, as well as other important methodological considerations^25^. For example, Keller^26^ illustrates the importance of accounting for confounding effects of other two-way interaction terms (e.g., G × covariate, E × covariate), which can complicate the interpretation of regression-based GxE analyses.

Both cannabis use and CUDs are complex genetic traits, influenced by multiple genetic variants of small effect (i.e., polygenicity) and aspects of the psychosocial environment (e.g., religiosity, trauma), which act independently and in concert. However, this information has not been effectively incorporated into genetic association studies. Polygenic risk scores (PRS), which aggregate genetic variants to reflect the underlying structure of complex traits, have been increasingly used to model the genetic architecture of substance use disorders^12,13,27–30^. In addition to the potential for identifying genetic risk markers for cannabis involvement^31,32^, these methods enable researchers to identify potential psychosocial mechanisms that modify genetic associations with CUD. Given that current findings from psychiatric genomics research are not yet at the point of clinical utility, identifying modifiable psychosocial risk and protective factors may be useful for downstream use in clinical and translational sciences.

Despite the number of studies that examine whether adverse experiences or religiosity moderate genetic influences on other substance use behavior (i.e., alcohol^33^, nicotine^13^), no published GxE studies examine moderation of genetic influences on CUD employing PRS derived from large, recent genetic studies of cannabis behavior. In this study, we use results from the Pasman et al. meta-analysis of cannabis use to examine (1) whether genetic propensity to cannabis use extends to cannabis problems as measured by the DSM-5, and (2) whether two well-established psychosocial factors known to influence substance use behavior, frequency of religious service attendance and trauma exposure, moderate polygenic influences on cannabis use and DSM-5 CUD symptoms in an independent sample enriched with individuals at high risk for substance use disorders.

## Methods

### Sample

The Collaborative Study on the Genetics of Alcoholism (COGA) is a large family-based sample enriched for alcohol and other substance use disorders^34^. COGA recruited alcohol-dependent probands from inpatient and outpatient AUD treatment facilities and comparison subjects from the same communities. Probands and their family members were interviewed with the Semi Structured Assessment of Alcoholism (SSAGA^35,36^). Informed consent was obtained from all participants and institutional review boards at all sites approved the study. For further details, see ref. ^34^. The primary analytic sample included 7591 individuals of EA, because the GWAS from which PRS was calculated was based on EA. Secondary analyses included 3359 individuals of African ancestry (AA). A description of the analytic sample, including self-reported “race/ethnicity” (correlation with genetic ancestry = 0.95, *p* < 0.001) is in Table 1.

**Table 1:** Description of the analytic sample, a subset of the Collaborative Study on the Genetics of Alcoholism

	All participants (*N*: 10,954)	European ancestry (*N*: 7599)	African ancestry (*N*: 3355)
Female	5805	3998	1807
Age at most recent interview	Range: 12–91 years; *M*: 35.61 (SD: 14.18)	Range: 12–91 years; *M*: 36.77 (SD: 14.83)	Range: 12–84 years; *M*: 32.88 (SD: 12.16)
Self-reported race			
White/Caucasian	7655	7454	201
Black/African American	2920	11	2908
Asian	29	11	18
Hispanic/Latino	619	207	412
Native American/American Indian/Pacific Islander/Other (non-specified)	357	127	230
Unknown	3	1	2
Trauma exposure	1443	847	596
Non-assaultive	1207	703	504
Assaultive	714	383	331
Sexually assaultive	264	165	99
Frequency of religious service attendance during 12 months prior to interview	Range: 0–356 days; *M*: 16.22 (SD: 33.89)	Range: 0–356 days; *M*: 15.88 (SD: 33.74)	Range: 0–260 days; *M*: 17.50 (SD: 34.89)
Cannabis use	7546	5014	2532
DSM-5 cannabis use disorder symptom count	Range: 0–11; *M*: 1.69 (SD: 2.83)	Range: 0–11; *M*: 1.58 (SD: 2.78)	Range: 0–11; *M*: 1.92 (SD: 2.90)

### Genotyping and quality control

Genotyping of COGA samples were performed at (1) the Center for Inherited Disease Research (1M array^37^), (2) Genome Technology Access Center at Washington University School of Medicine (Illumina OmniExpress^38^, and (3) Rutgers University (Affymetrix Smokescreen array^39^). All A/T and C/G SNPs were removed and a common set of approximately 47,000 SNPs was used to detect duplicate samples and to revise the reported pedigree structure. Family structures were altered as needed, and SNP genotypes were tested for Mendelian inconsistencies^40^ within the revised family structure. Genotype inconsistencies were set to missing. Imputation was to 1000 Genomes (Phase 3, version 5) using SHAPEIT^41^ and then Minimac3^42^ and performed separately within data from the three genotyping sites. Only SNPs with a genotype rate >75% were included in analyses. Further details on the genotyping arrays, samples, and processing are in Supplementary Information. To maximize the utility of COGA’s family-based design, participants were assigned a family-based ancestry of EA or AA, according to the majority of individual-based ancestry in that family, based on principal components (PCs) derived from GWAS data. In the <10% of cases of equal proportion (e.g., 50% EA and 50% AA), the family was assigned the more heterogeneous ancestry (AA). All analyses were conducted separately by ancestry, with ancestral PCs included as covariates in all PRS analyses.

### Genome-wide cannabis use PRS

We used genome-wide association estimates for cannabis ever use from the 2018 Pasman et al.^9^ meta-analysis of GWAS data from the ICC and UK Biobank for ever use of cannabis during one’s lifetime to calculate a cannabis ever use PRS for participants in COGA. We used the -score procedure in PLINK^43^, which computes a linear function of the number of scored alleles an individual possesses weighted by the associated GWAS beta coefficient. Matching SNPs were pruned for linkage disequilibrium (LD) based on 1000 Genome phase 3 reference panel genotype data for EA for EA participants and AA for AA participants, with clumping based on the Pasman et al. GWAS *p* values using a 500-kb physical distance and an LD threshold of *r*^2^ ≥ 0.25. We note that the Pasman et al. study^9^ is entirely of EA. Because of this, genetic influences captured in this PRS will likely predict cannabis use behavior in AA samples poorly^44^. Therefore, the current manuscript focuses on COGA’s EA participants. However, COGA includes one of the largest genetically informative AA samples available. We therefore conducted a series of exploratory analyses in 3359 AA participants to test the predictive power of EA-based summary statistics in an AA population (Supplementary Table 2). Secondary analyses conducted in the AA participants followed the same procedures as described for EA participants. We calculated a series of scores in our sample that included SNPs meeting increasingly stringent *p* value thresholds (*p* < 0.0001, *p* < 0.001, *p* < 0.01, *p* < 0.05, *p* < 0.10, *p* < 0.20, *p* < 0.30, *p* < 0.40, *p* < 0.50) in the discovery GWAS.

### Measures

Cannabis use was assessed with the following question: “Have you ever used marijuana or hashish?” Response options were Yes (1) or No (0). DSM-5 CUD symptoms were derived from the SSAGA and included 0–11 symptoms, including: tolerance, withdrawal, activities given up due to cannabis use, time dominated by cannabis use, cannabis use despite physical or psychological consequences, craving, etc. Among individuals with more than one interview, data from the most recent interview with the maximum number of CUD symptoms was used.

Frequency of religious service attendance was assessed with the following question: In the past 12 months, how many times did you attend religious services? Response options ranged from 0 to 365. Values were log-transformed. Sensitivity analyses also tested “any attendance” (0 vs. other values) and “weekly” attendance (values ≥52 vs. values <52), and models stratified by attendance before and after age 18 years. In addition, participants were asked about their religious preference and given the following response options: Catholic, Protestant (e.g., non-fundamentalist Baptist, Episcopalian, Lutheran, Methodist, Presbyterian), Fundamentalist Protestant (e.g., Assembly of God, fundamentalist Baptist, Brethren, Evangelical Christian, Foursquare Gospel Church, Jehovah’s Witness, Nazarene, Pentecostal), Jewish, Moslem, Buddhist, Not affiliated, Agnostic, Atheist, Other (e.g., B’hai, Hindu, Unitarian, Wicca), Greek-, Serbian-, Russian-Orthodox, Christian, other (e.g., Charismatic, Christian Scientist, Mennonite, Mormon, Seventh Day Adventist), Christian, not otherwise specified.

Two different SSAGA assessments were used to ascertain trauma exposure data in the current study. In all, 58.6% (*N*: 3875) of the EA analytic sample had trauma assessed using the SSAGA-IV^35^ as described previously^45^. Briefly, the SSAGA-IV post-traumatic stress disorder section begins by asking participants about “terrible, frightening, or horrible experiences” they may have had in their lifetime. Subsequently, they are asked about having ever experienced 21 specific potentially traumatic events, including assaultive, non-assaultive, and sexually assaultive exposures (detailed in Supplementary Table 3). Trauma exposure data on an additional 2738 participants was ascertained from earlier waves of the COGA study in which the SSAGA-II was used. The SSAGA-II asked participants if they “had ever experienced or witnessed something that is so horrible that it would be distressing or upsetting to almost anyone?” The SSAGA-II then listed some examples of potentially traumatic events and asked the participant to list up to three events. In order to use available trauma exposure data from both the SSAGA-IV and SSAGA-II, we examined a binary measure of trauma exposure (0: not exposed, 1: exposed), based on SSAGA-II and SSAGA-IV responses, to maximize statistical power, which is especially crucial in GxE studies. All individuals who were not interviewed using the SSAGA-II or SSAGA-IV and hence were not asked about trauma exposure were coded as “unknown” and excluded from PRS × trauma analyses. As compared to those with available trauma data (*N*: 6613), those with missing data related to trauma exposure (“unknown”, *N*: 4341) were more likely to be older and “White/Caucasian.”

### Statistical analysis

In independent models, cannabis use ever (0: never, 1: ever use) and DSM-5 CUD symptom counts (0–11 symptoms, log-transformed) were regressed using Poisson models on cannabis ever use PRS from the Pasman et al. study^9^. Models were adjusted for ancestral PCs (PC1–PC3), age (range: 12–91 years; mean = 36.77, SD: 14.83), birth cohort (dummy variables representing birth years prior to 1930, 1930–1949, 1950–1969, and 1970 and after), and sex (0 = male, 1 = female), given prior evidence that cannabis use and problems differ across these groups^46^. In addition, genotype array was used as a covariate in all analyses since different platforms were used across the sample (detailed above). In addition, we accounted for the nested nature of the family-based sample in all regression analyses using hierarchical multi-level modeling. A Bonferroni correction for multiple testing, adjusting for two cannabis-related outcomes and nine PRS thresholds was applied to all findings, requiring a *p* value < 0.003 to meet criteria for statistical significance. Cannabis use and CUD were moderately correlated (*r*^2^: 0.40, *p* < 0.001); PRS across varying thresholds were only modestly correlated (*r*^2^ ranged from 0.04 to 0.07). To further reduce the multiple test burden, moderation analyses were only conducted with those PRS for which a significant main effect on cannabis use and/or CUD symptom count (CUDsx) was observed. When significant main effects of the PRS were observed, we tested for multiplicative interactions with trauma exposure and frequency of religious service attendance. When significant main effects of the PRS were observed, we tested for multiplicative interactions with trauma exposure and frequency of religious service attendance. Note that only individuals who reported ever using cannabis in their lifetime were included in DSM-5 CUDsx analyses. All other individuals were classified as unknown and were not included in analyses of CUD symptoms. Sensitivity analyses including these individuals were conducted as well to examine the influence of excluding these individuals in our analyses. In addition, secondary models included cross-terms for all variables included in the interaction models (e.g., Model 2 included PRS × trauma, PRS × age, PRS × sex, PRS × birth cohort, PRS × PCs 1–3, trauma × age, trauma × sex, trauma × birth cohort, trauma × PCs 1–3, etc.) as suggested by Keller^26^.

## Results

Of the primary analytic sample (i.e., EA participants with GWAS data, *n* = 7591), 66.3% had ever used cannabis in their lifetime, and 28.2% met criteria for lifetime DSM-5 CUD [mild (2–3 criteria; 8.8%), moderate (4–5 criteria; 6.9%), severe (6+ criteria; 12.4%)]. Among those who had ever used cannabis in their lifetime, 42.6% met criteria for lifetime DSM-5 CUD [mild (13.6%), moderate (10.6%), severe (18.4%)]. Among cannabis users, mean number of CUD symptoms was 2.39 (Range: 0–11, SD: 3.14). A greater number of symptoms were observed among men and younger individuals.

**Fig. 1:**
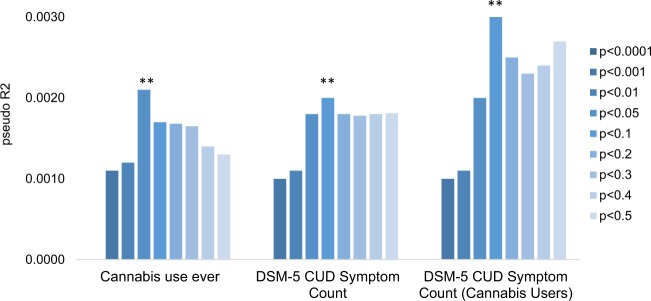
Main effects of cannabis ever use polygenic risk scores on cannabis use and DSM-5 cannabis use disorder symptom count in COGA participants of European ancestry. Double asterisks (**) denote associations that withstand a multiple test correction

Among EAs, main effects of the PRS were observed for cannabis ever use and for DSM-5 CUDsx (Table 2; Fig. 1): PRS with *p* < 0.05, *p* < 0.10, *p* < 0.20, *p* < 0.30, *p* < 0.40, and *p* < 0.50 thresholds were nominally associated with ever cannabis use (*p* ranging from 0.009 to 0.001) and PRS with *p* < 0.001, *p* < 0.01, *p* < 0.05, *p* < 0.10, *p* < 0.20, *p* < 0.30, *p* < 0.40, and *p* < 0.50 thresholds were associated with DSM-5 CUDsx (*p* ranging from 0.036 to 0.001), but only nominally in ever users (*p* ranging from 0.043 to 0.005). Associations of PRS (*p* < 0.05 threshold) with ever cannabis use and PRS (*p* < 0.10 threshold) with DSM-5 CUDsx withstood a Bonferonni correction for multiple testing. Above and beyond the aggregate effects of the sex, birth cohort, genotype array, and ancestral PCs, the PRS with *p* < 0.05 accounted for 0.2% of the variance in ever cannabis use (area under the curve (AUC): 0.53 (0.52, 0.54)) and 0.2% of the variance in DSM-5 CUDsx (DSM-5 CUD diagnosis AUC: 0.51 (0.48, 0.55)) (Table 2). No main effects of the PRS were observed for ever use of cannabis or for DSM-5 CUDsx among AA participants (Supplementary Table 2).

**Table 2:** Main effects of cannabis initiation polygenic risk scores on cannabis use and DSM-5 cannabis use disorder symptom count in COGA participants of European ancestry

Threshold	Cannabis use ever (lifetime)			DSM-5 cannabis use disorder symptom count			DSM-5 cannabis use disorder symptom count (among cannabis users)		
	*R* ^2^	Beta	*p* value	*R* ^2^	Beta	*p* value	*R* ^2^	Beta	*p* value
*p* < 0.0001	0.000	0.018	0.200	0.000	0.004	0.769	0.000	0.015	0.428
*p* < 0.001	0.001	0.029	0.050	0.001	0.032	0.036	0.001	0.037	0.059
*p* < 0.01	0.001	0.028	0.050	0.000	0.019	0.222	0.000	0.023	0.249
***p*** **<** **0.05**	**0.002**	**0.046**	0.001**	**0.002**	**0.041**	**0.007**	**0.002**	**0.039**	**0.043**
***p*** **<** **0.1**	**0.001**	**0.039**	**0.006**	**0.002**	**0.050**	0.001**	**0.003**	**0.055**	**0.005**
***p*** **<** **0.2**	**0.001**	**0.039**	**0.007**	**0.001**	**0.040**	**0.010**	**0.002**	**0.041**	**0.038**
***p*** **<** **0.3**	**0.001**	**0.042**	**0.004**	**0.001**	**0.040**	**0.011**	**0.002**	**0.042**	**0.037**
***p*** **<** **0.4**	**0.001**	**0.040**	**0.006**	**0.002**	**0.041**	**0.009**	**0.002**	**0.044**	**0.029**
***p*** **<** **0.5**	**0.001**	**0.038**	**0.009**	**0.002**	**0.041**	**0.009**	**0.002**	**0.045**	**0.025**

Note: Polygenic risk scores are derived from Pasman et al.’s cannabis initiation GWAS^4^ summary statistics. Covariates include sex, age, birth cohort, genotype array, PCs 1–3. Boldface indicates estimates that are statistically significant with *p* < 0.05; double asterisks (**) indicate estimates that withstand Bonferroni test correction (0.05/27 = 0.0018)

### Trauma exposure

**Fig. 2:**
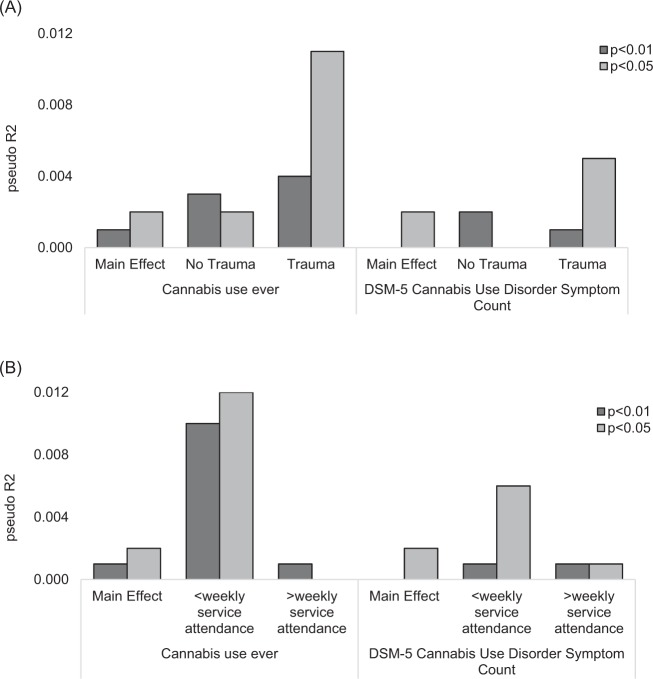
Main effects of PRS (*p* < 0.01 and *p* < 0.05 *p* value thresholds) and PRS effects stratified by trauma exposure (no trauma = not exposed to a traumatic event; trauma = exposed to a traumatic event) and frequency of weekly religious service attendance. Main effects of PRS (*p* < 0.01 and *p* < 0.05 *p* value thresholds) and PRS effects stratified by trauma exposure (no trauma = not exposed to a traumatic event; trauma = exposed to a traumatic event) are displayed in **a**. Main effects of PRS (*p* < 0.01 and *p* < 0.05 *p* value thresholds) and PRS effects stratified by frequency of weekly religious service attendance are displayed in **b**. PRS had a greater influence on cannabis ever use and DSM-5 CUD symptom count among those who had been exposed to traumatic events (**a**) and among those who less frequently attended religious services (**b**)

Data on trauma exposure was available for analysis on 6613 EA individuals. Lifetime trauma exposure was reported by 12.8% of the sample. Note, trauma exposure was reported by 12.3% of those assessed with the SSAGA-IV (full trauma checklist, Supplementary Table 3) and 15.4% of those assessed with an earlier SSAGA (abbreviated, as described above). Trauma exposure was associated with cannabis use (*B*: 0.065, *p* < 0.017, *R*^2^: 0.004) and DSM-5 CUDsx (*B*: 0.161, *p* < 0.0001, *R*^2^: 0.026); trauma exposure was more common in cannabis users (12.9%) compared with non-users (7.6%) and in those meeting criteria for DSM-5 CUD (15.8%). Trauma exposure also moderated the association of the PRS (*p* < 0.05 threshold) with cannabis ever use in *both* Model 1, which adjusted for age, sex, birth cohort, genotype array, and genetic ancestry, and Model 2, which also included two-way interactions for all variables included in the model (Table 3). Interactions among trauma exposure and PRS with *p* < 0.05 and *p* < 0.10 thresholds were observed for cannabis ever use but not DSM-5 CUDsx (Table 3). Both PRS had a greater influence on cannabis ever use and DSM-5 CUDsx among those exposed to trauma (*R*^2^: 0.011–0.014; AUC: 0.58 (0.53, 0.62)) as compared to those who were not exposed to trauma (*R*^2^: 0.001–0.002; AUC: 0.49 (0.43, 0.54), Fig. 2a). Trauma exposure was not predicted by cannabis use PRS (*p* > 0.26).

**Table 3:** Moderation of cannabis use and DSM-5 CUD symptom count by trauma exposure and frequency of religious service attendance among COGA participants of European ancestry; results from Model 1 that includes the following covariates: age, sex, birth cohort, genotype array, and PCs 1–3

PRS threshold	Cannabis use ever (lifetime)			DSM-5 cannabis use disorder Sx count		
	*R* ^2^	Beta	*p* value	*R* ^2^	Beta	*p* value
Trauma exposure × PRS						
*p* < 0.0001	—	—	—	—	—	—
*p* < 0.001	—	—	—	—	—	—
*p* < 0.01	—	—	—	—	—	—
*p* < 0.05	**0.005**	**0.181**	**0.009** ^a^	0.001	0.098	0.148
*p* < 0.1	**0.004**	**0.341**	**0.016**	0.001	0.115	0.406
*p* < 0.2	—	—	—	—	—	—
*p* < 0.3	—	—	—	—	—	—
*p* < 0.4	—	—	—	—	—	—
*p* < 0.5	—	—	—	—	—	—
Frequency of service attendance × PRS						
*p* < 0.0001	—	—	—	—	—	—
*p* < 0.001	—	—	—	—	—	—
*p* < 0.01	—	—	—	—	—	—
*p* < 0.05	**0.008**	**0.281**	**0.006**	0.004	0.194	0.061
*p* < 0.1	**0.007**	**0.448**	**0.014**	**0.008**	**0.476**	**0.010**
*p* < 0.2	—	—	—	—	—	—
*p* < 0.3	—	—	—	—	—	—
*p* < 0.4	—	—	—	—	—	—
*p* < 0.5	—	—	—	—	—	—

Moderation analyses were only conducted on scores where a statistically significant (*p* < 0.002) main effect was observed. Boldface indicates estimates with *p* value < 0.05

^a^Denotes results that were statistically significant in Model 2, which includes the following covariates: age, sex, birth cohort, PCs1–3, PRS × trauma/frequency of service attendance, PRS × age, PRS × sex, PRS × PCs 1–3, trauma/frequency of service attendance × age, trauma/frequency of service attendance × sex, trauma × PCs 1–3. Data from Model 2 are displayed in Supplementary Table 1

### Religiosity

In all, 66.5% of the full sample reported *any* attendance at religious services in the past 12 months. While 29% of the analytic sample did not specify their religious affiliation, and 19.7% reported “no affiliation, agnostic, or atheist,” 18.9% reported their affiliation as Christian, 15.3% Catholic, 9.7% Protestant, and 4.8% Fundamentalist Protestant. All other affiliations (Jewish, Moslem, Buddhist, etc.) were reported by <1% of the sample. Within the analytic sample, 12.6% of participants reported that they affiliated with a religion that had rules forbidding alcohol use; these participants were most likely to identify as Christian (broadly), followed by Protestant or Fundamentalist Protestant and Buddhist. The mean number of days of service attendance was 15.9 (SD: 33.74) days within the 12 months prior to the interview. Both cannabis ever use (*B*: −0.185, *p* < 0.001, *R*^2^: 0.052) and DSM-5 CUDsx (*B*: −0.099, *p* < 0.001, *R*^2^: 0.058) were associated with frequency of religious service attendance; any service attendance was less common in cannabis users (61.9%) than in non-users (81.0%) and in those meeting criteria for DSM-5 CUD (50.5%). Similar patterns were observed for *any* attendance (cannabis use: *B*: −0.124, *p* < 0.001, *R*^2^: 0.015; CUDsx: *B*: −0.136, *p* < 0.001, *R*^2^: 0.018) and *weekly* attendance (cannabis use: *B*: −0.214, *p* < 0.001, *R*^2^: 0.045; CUDsx: *B*: −0.135, *p* < 0.001, *R*^2^: 0.018). Frequency of religious service attendance moderated the association of the PRS with cannabis ever use and DSM-5 CUDsx but in the models that adjusted for age, sex, birth cohort, and ancestral PCs *only* (Model 1, Table 3). For cannabis ever use, interactions were observed between frequency of religious service attendance and PRS (*p* < 0.05 and *p* < 0.10 thresholds), and for DSM-5, interaction was observed between frequency of religious service attendance and PRS (*p* < 0.10 threshold). All PRS had a greater influence on cannabis ever use and DSM-5 CUDsx among those who less frequently attended religious services as compared to those who more frequently attended services (Fig. 2b). However, in models that also included cross-terms for all variables in the interaction model, no significant moderation effects were observed (Model 2, Table 3). Post hoc step-wise models indicated that the interaction among frequency of service attendance and the PRS was diminished by the inclusion of sex × religious service attendance frequency (however, the PRS by service attendance frequency interaction remained statistically significant, *p* < 0.037). The inclusion of the ancestral PC1 by religious service attendance frequency interaction significantly reduced the variance explained by this model (*p* > 0.30). Sensitivity analyses were conducted stratified by age (above and below age 18 years) to determine whether effects differed by age; similar results were observed for those under and over age 18 years. Religious service attendance was unrelated to cannabis use PRS (*p* > 0.14). However, the first genetic ancestral component (PC1) was positively correlated with Jewish religious affiliations (*r*: 0.39, *p* < 0.0001) and negatively correlated with Protestant religious affiliations (*r*: −0.14, *p* < 0.0001).

## Discussion

The current study demonstrates that polygenic liability to cannabis use was nominally associated with cannabis use (ever) and with DSM-5 CUDsx in COGA, a deeply phenotyped family sample enriched for individuals with substance use disorders. In addition, moderation of this polygenic risk for cannabis use was observed with one well-known risk factor, trauma exposure. Weaker evidence of moderation by frequency of religious service attendance was also observed. These findings provide the first evidence that trauma may potentiate polygenic risk for CUDs, while for some groups, religious service attendance may serve to dampen genetic susceptibility.

Our study extends the GxE literature on trauma exposure and cannabis use substantially. Previous studies have shown that different types of trauma exposure (physical assault, sexual assault, etc.) significantly increase risk for CUD^20,46^ and that adverse experiences exacerbate genetic influences on alcohol and drug use behaviors^47^. To our knowledge, this is among the first studies to document the role of lifetime trauma in accentuating polygenic risk to cannabis use. Our results indicate that the influence of polygenic risk on cannabis use accounts for ~1–2% of the variance in CUD symptoms among those who are exposed to trauma as compared with 0.1–0.2% among those who are not exposed to trauma. Future genetic studies with larger sample sizes should examine the PRS × trauma exposure effects on substance use behavior considering specific types of trauma, age of trauma exposure, and duration and frequency of trauma exposure to gain further understanding of these findings.

Consistent with previous studies^48^, we observed that individuals who more frequently attended religious services were less likely to use cannabis in their lifetime and endorsed fewer DSM-5 CUD symptoms. Epidemiological research has identified several aspects of religiosity as important protective factors against substance use problems. For example, religious service attendance provides both interpersonal and community social support that may serve to increase resiliency and limit expression of genetic propensity to cannabis use. In addition, protective effects of religion may be mediated by the degree to which specific religions foster restrictive social norms and attitudes regarding substance use^49^. In this study, 12.6% of participants reported that they affiliated with a religion that had rules forbidding alcohol use; these participants were most likely to identify as Christian (broadly), followed by Protestant or Fundamentalist Protestant and Buddhist. We note, however, that, while there may be religious proscriptions regarding alcohol use, these may or may not extend to cannabis use. Further, some twin studies have shown that aspects of religiosity moderate genetic influences on alcohol use behavior^19–22^. For example, a Dutch twin study found that, among women without a religious upbringing, genetic influences accounted for 40% of the variance in alcohol use ever compared to 0% among women raised religiously. Conversely, church attendance was not a moderator of genetic influences on adolescent alcohol consumption among adult male twins from Virginia, suggesting that findings may differ as a function of sex and culture. The current study expands this literature by providing evidence that the influence of cannabis use PRS on cannabis use and DSM-5 CUD symptoms is moderated by frequency of religious service attendance in a cohort of males and females between ages 12 and 91 years. We note, however, that none of these findings withstood a multiple test correction. Interestingly, while studies have shown that religious service attendance during adulthood may be a more robust safeguard against heavy substance use and may reflect innate tendencies toward religious affiliations, relative to childhood attendance that bears a strong familial component^45^, our findings did not differ significantly when analyses were stratified by age. In addition, since social support (outside of the religious context) has also been identified as an important protective factor against alcohol and other drug use^50^ and has been shown to diminish genetic risk for other substance use problems^13^, social and community aspects of religiosity, likely captured by frequency of religious service attendance, may be an important moderator of cannabis use.

Keller notes that ignoring two-way interactions between the environmental measure and other covariate terms in GxE analyses can generate spurious findings^42^. Upon addition of these two-way terms, the moderating effect of religious service attendance was diminished by the inclusion of interactions between service attendance frequency and the first ancestral PC and with sex. Interestingly, within the EA analytic sample, PC1 is significantly positively correlated with Jewish religious affiliations (*r*: 0.39, *p* < 0.0001) and negatively correlated with Protestant (non-fundamentalist Baptist, Episcopalian, Lutheran, Methodist, Presbyterian) religious affiliations (*r*: −0.14, *p* < 0.0001). The influence of ancestral PC1 is likely reflecting both cultural differences regarding cannabis use by ancestral background (i.e., country of origin) within European Americans and differences by religious affiliation in attitudes toward cannabis use demonstrated by previous studies^48,51,52^. In addition, religious service attendance was a more prominent buffer from CUD among women relative to men, which is supported by previous studies that have shown the inverse associations of religiosity and substance use behavior are greater among women relative to men^48,51,52^.

There are several strengths of this study. We demonstrated polygenic effects of common genetic variation on cannabis use, with weaker associations observed with persistence into DSM-CUD symptoms, in a well-characterized sample enriched for substance use problems. This provides molecular polygenic support for some genetic distinction of cannabis use and CUD demonstrated in several twin studies^3^. In addition, we used a relatively novel approach by testing GxE using polygenic scores, which allowed a focus on how polygenic influences on cannabis involvement differ as a function of psychosocial context. Further, we responded to critiques of GxE studies, which largely do not account for potential confounding of GxE effects^25,26^. Specifically, modeling of cross-term interactions revealed important influences of ancestry and sex on the PRS × frequency of religious service attendance effects observed in the current study. It should be noted that, while this study utilized genetic data to assign ancestry, which in our analytic sample was highly correlated with self-reported “race/ethnicity” (*r*: 0.95, *p* < 0.001), these are distinct constructs both shown to influence risk of substance use-related health outcomes, namely, through interactions with the psychosocial environment. In our data, the addition of self-reported “race/ethnicity” does not provide additional explanatory value, over and above genetic ancestry. Future PolyGxE studies should consider the role of other important social and cultural constructs.

Despite its strengths, our results should be interpreted within the context of the following limitations. The present analysis uses retrospective reports of substance use and traumatic life events and relies on measures of past year religiosity. The temporality of associations observed in this study is ambiguous; that is, the timing of traumatic exposures and religious service attendance with regard to cannabis use behaviors was not considered in the present study. While this study has largely focused on interpretations that assume trauma exposure and religious service attendance preceded cannabis use problems, previous studies show that substance use and problems may also precede both trauma exposure and religious service practices. Timing of traumatic exposure, religious service attendance, and cannabis use behaviors should be used to further understanding of the temporality of these associations. In addition, this study focused on participants of EA due to the ancestral composition of the discovery cohorts in the Pasman et al. study^9^. While congruent with what is expected for PRS derived from GWASs with sample sizes and effect sizes similar to those observed in Pasman et al.^9^, predictive power of the PRS observed in this study was poor or non-significant for CUDsx. Given the reliance of PRS on large, well-powered samples, future studies should re-examine the relations examined in this study with larger studies that will soon be available to the research community. In addition, GWAS that specifically examine problematic cannabis use may be needed to achieve predictive utility for CUD. Finally, these findings may not be generalizable to individuals of other ancestral backgrounds as seen in our AA cohort (Supplementary Table 2). While Supplementary Table 2 shows the prediction of cannabis use and CUDsx in COGA’s participants of AA, we note the limitations of applying EA-derived summary statistics to participants of AA. Large discovery GWAS in non-Europeans could be foundational in bridging the disparity in PRS research that is heavily weighted toward European populations even though negative consequences of addictions disproportionately impact those from other ancestral backgrounds^53^.

Although evidence for GxE on substance use behavior has been well established, translation of this research into useful risk-factor targets for public health and clinical intervention is lacking. Among the reasons is the complex etiology of cannabis use and problems, which involves multiple genetic and psychosocial influences, each having very subtle effects. Despite this challenge, evidence for the influence of GxEs on substance use behaviors is mounting. In this study, we show that polygenic liability to cannabis ever use predicted cannabis use but not progression to DSM-5 CUDsx in COGA. However, this accounts for an extremely limited (∼0.2%) portion of the total variance in cannabis use. Among the trauma exposed, this increased to ~1.4% of the total variance in cannabis use. Given the limited predictive utility of current genetic information for cannabis use and CUD, it is important to note that both of the psychosocial contexts measured in this study explained a significantly greater portion of the variance in CUD as compared with the PRS (trauma exposure *R*^2^: 0.026; frequency of service attendance *R*^2^: 0.058). Findings from this study suggest that clinicians should pay particular attention to the possible escalation of cannabis use and misuse in those with genetic susceptibility and a history of trauma exposure. Treatment approaches that are tailored toward both prevention of CUD and ameliorating the burden of prior experienced trauma require continued care and monitoring. Encouragingly, regular engagement in religious services, at least within some communities, is likely to dampen the influence of genetic vulnerability. It is likely that other forms of prosocial engagement perform a similar protective function. Therefore, prevention strategies that encourage such pro-social engagement is warranted in vulnerable populations, such as those exposed to trauma. Though we note that alternative explanations for this study’s findings (e.g., confounding by sex and/or ethnicity, the influence of cannabis use, and problems on religiosity) cannot be ruled out.

In conclusion, polygenic liability to cannabis use was nominally associated with cannabis ever use and with progression to DSM-5 CUDsx in an independent sample of individuals enriched for substance use problems. In addition, PRSxE was observed with trauma exposure, with less consistent moderation effects observed with religious service attendance that are driven by important interactions with ancestry and sex. These findings provide the first evidence of PRSxE effects for cannabis use and support previous findings that trauma exacerbates genetic risk for substance use, while religious service attendance may serve as a protective factor. This study also demonstrates that, by not modeling important aspects of the psychosocial environment, we may mask genetic influences on polygenic traits, such as cannabis use.

## Supplementary information

Supplementary Table Captions

Supplementary Table 1

Supplementary Table 2

Supplementary Table 3

## References

[1] Hasin DS (2016). Prevalence and correlates of DSM-5 cannabis use disorder, 2012-2013: findings from the National Epidemiologic Survey on Alcohol and Related Conditions–III. Am. J. Psychiatry.

[2] Grucza RA, Agrawal A, Krauss MJ, Cavazos-Rehg PA, Bierut LJ (2016). Recent trends in the prevalence of marijuana use and associated disorders in the United States. JAMA Psychiatry.

[3] Agrawal A, Lynskey MT (2006). The genetic epidemiology of cannabis use, abuse and dependence. Addiction.

[4] Stringer S (2016). Genome-wide association study of lifetime cannabis use based on a large meta-analytic sample of 32 330 subjects from the International Cannabis Consortium. Transl. Psychiatry.

[5] Shanahan MJ, Hofer SM (2005). Social context in gene-environment interactions: retrospect and prospect. J. Gerontol. Ser. B Psychol. Sci. Soc. Sci..

[6] Sherva R (2016). Genome-wide association study of cannabis dependence severity, novel risk variants, and shared genetic risks. JAMA Psychiatry.

[7] Agrawal, A. et al. Genome-wide association study identifies a novel locus for cannabis dependence. *Mol. Psychiatry* **23**, 1293–1302 (2018). 10.1038/mp.2017.200 PMC5938138 29112194

[8] Demontis, D. et al. Genome-wide association study implicates CHRNA2 in cannabis use disorder. *Nat. Neurosci.* **22**, 1066–1074 (2019). 10.1038/s41593-019-0416-1 PMC7596896 31209380

[9] Pasman, J. A. et al. Genome-wide association analysis of lifetime cannabis use (N = 184,765) identifies new risk loci, genetic overlap with mental health, and a causal influence of schizophrenia on cannabis use. *Nat. Neurosci.* **21**, 1161–1170 (2019). 10.1038/s41593-018-0206-1 PMC6386176 30150663

[10] Edenberg HJ, McClintick JN (2018). Alcohol dehydrogenases, aldehyde dehydrogenases, and alcohol use disorders: a critical review. Alcohol. Clin. Exp. Res..

[11] Kendler KS, Myers J, Dick D, Prescott CA (2010). The relationship between genetic influences on alcohol dependence and on patterns of alcohol consumption. Alcohol. Clin. Exp. Res..

[12] Salvatore JE (2014). Polygenic scores predict alcohol problems in an independent sample and show moderation by the environment. Genes (Basel).

[13] Meyers J L, Cerdá M, Galea S, Keyes K M, Aiello A E, Uddin M, Wildman D E, Koenen K C (2013). Interaction between polygenic risk for cigarette use and environmental exposures in the Detroit neighborhood health study. Translational Psychiatry.

[14] Koopmans JR, Slutske WS, van Baal GC, Boomsma DI (1999). The influence of religion on alcohol use initiation: evidence for genotype X environment interaction. Behav. Genet..

[15] Button TMM, Hewitt JK, Rhee SH, Corley RP, Stallings MC (2010). The moderating effect of religiosity on the genetic variance of problem alcohol use. Alcohol. Clin. Exp. Res..

[16] Harden KP (2010). Does religious involvement protect against early drinking? A behavior genetic approach. J. Child Psychol. Psychiatry.

[17] Timberlake DS (2006). The moderating effects of religiosity on the genetic and environmental determinants of smoking initiation. Nicotine Tob. Res..

[18] Dick DM, Kendler KS (2012). The impact of gene-environment interaction on alcohol use disorders. Alcohol Res..

[19] Kristjansson S (2016). The variance shared across forms of childhood trauma is strongly associated with liability for psychiatric and substance use disorders. Brain Behav..

[20] Carliner H (2016). Childhood trauma and illicit drug use in adolescence: a population-based national comorbidity survey replication-adolescent supplement study. J. Am. Acad. Child Adolesc. Psychiatry.

[21] Meyers Jacquelyn L., Sartor Carolyn E., Werner Kimberly B., Koenen Karestan C., Grant Bridget F., Hasin Deborah (2018). Childhood interpersonal violence and adult alcohol, cannabis, and tobacco use disorders: variation by race/ethnicity?. Psychological Medicine.

[22] Grant JD (2017). Phenotypic and familial associations between childhood maltreatment and cannabis initiation and problems in young adult European-American and African-American women. Drug Alcohol Depend..

[23] Meyers JL (2015). Childhood adversity moderates the effect of ADH1B on risk for alcohol-related phenotypes in Jewish Israeli drinkers. Addict. Biol..

[24] Sartor CE, Wang Z, Xu K, Kranzler HR, Gelernter J (2014). The joint effects of ADH1B variants and childhood adversity on alcohol related phenotypes in African-American and European-American women and men. Alcohol. Clin. Exp. Res..

[25] Duncan LE, Keller MC (2011). A critical review of the first 10 years of candidate gene-by-environment interaction research in psychiatry. Am. J. Psychiatry.

[26] Keller MC (2014). Gene × environment interaction studies have not properly controlled for potential confounders: the problem and the (simple) solution. Biol. Psychiatry.

[27] Vink JM (2014). Polygenic risk scores for smoking: predictors for alcohol and cannabis use?. Addiction.

[28] Salvatore JE (2015). Polygenic risk for externalizing disorders: gene-by-development and gene-by-environment effects in adolescents and young adults. Clin. Psychol. Sci..

[29] Musci RJ, Uhl G, Maher B, Ialongo NS (2015). Testing gene × environment moderation of tobacco and marijuana use trajectories in adolescence and young adulthood. J. Consult. Clin. Psychol..

[30] Musci RJ (2018). Polygenic score × intervention moderation: an application of discrete-time survival analysis to model the timing of first marijuana use among urban youth. Prev. Sci..

[31] Salvatore JE (2018). Incorporating functional genomic information to enhance polygenic signal and identify variants involved in gene-by-environment interaction for young adult alcohol problems. Alcohol. Clin. Exp. Res..

[32] Savage JE (2018). Polygenic risk score prediction of alcohol dependence symptoms across population-based and clinically ascertained samples. Alcohol. Clin. Exp. Res..

[33] Young-Wolff KC, Enoch M-A, Prescott CA (2011). The influence of gene-environment interactions on alcohol consumption and alcohol use disorders: a comprehensive review. Clin. Psychol. Rev..

[34] Begleiter H (1999). Description of the Genetic Analysis Workshop 11 Collaborative Study on the Genetics of Alcoholism. Genet. Epidemiol..

[35] Bucholz KK (1994). A new, semi-structured psychiatric interview for use in genetic linkage studies: a report on the reliability of the SSAGA. J. Stud. Alcohol.

[36] Kuperman S (2013). A model to determine the likely age of an adolescent’s first drink of alcohol. Pediatrics.

[37] Edenberg HJ (2010). Genome-wide association study of alcohol dependence implicates a region on chromosome 11. Alcohol. Clin. Exp. Res..

[38] Wang J-C (2013). A genome-wide association study of alcohol-dependence symptom counts in extended pedigrees identifies C15orf53. Mol. Psychiatry.

[39] Baurley JW, Edlund CK, Pardamean CI, Conti DV, Bergen AW (2016). Smokescreen: a targeted genotyping array for addiction research. BMC Genomics.

[40] O’Connell JR, Weeks DE (1998). PedCheck: a program for identification of genotype incompatibilities in linkage analysis. Am. J. Hum. Genet..

[41] Delaneau O, Howie B, Cox AJ, Zagury J-F, Marchini J (2013). Haplotype estimation using sequencing reads. Am. J. Hum. Genet..

[42] Das S (2016). Next-generation genotype imputation service and methods. Nat. Genet..

[43] Purcell S (2007). PLINK: a tool set for whole-genome association and population-based linkage analyses. Am. J. Hum. Genet..

[44] Martin AR (2017). Human demographic history impacts genetic risk prediction across diverse populations. Am. J. Hum. Genet..

[45] McCutcheon Vivia V., Agrawal Arpana, Kuo Sally I-Chun, Su Jinni, Dick Danielle M., Meyers Jacquelyn L., Edenberg Howard J., Nurnberger John I., Kramer John R., Kuperman Samuel, Schuckit Marc A., Hesselbrock Victor M., Brooks Andrew, Porjesz Bernice, Bucholz Kathleen K. (2017). Associations of parental alcohol use disorders and parental separation with offspring initiation of alcohol, cigarette and cannabis use and sexual debut in high-risk families. Addiction.

[46] Bucholz Kathleen K., McCutcheon Vivia V., Agrawal Arpana, Dick Danielle M., Hesselbrock Victor M., Kramer John R., Kuperman Samuel, Nurnberger John I., Salvatore Jessica E., Schuckit Marc A., Bierut Laura J., Foroud Tatiana M., Chan Grace, Hesselbrock Michie, Meyers Jacquelyn L., Edenberg Howard J., Porjesz Bernice (2017). Comparison of Parent, Peer, Psychiatric, and Cannabis Use Influences Across Stages of Offspring Alcohol Involvement: Evidence from the COGA Prospective Study. Alcoholism: Clinical and Experimental Research.

[47] Enoch M-A (2010). The influence of GABRA2, childhood trauma, and their interaction on alcohol, heroin, and cocaine dependence. Biol. Psychiatry.

[48] Meyers, J. L., Brown, Q., Grant, B. F. & Hasin, D. Religiosity, race/ethnicity, and alcohol use behaviors in the United States. *Psychol. Med*. **47**, 103–114 (2017). 10.1017/S0033291716001975 PMC5319200 27667499

[49] Haber JR (2013). Religion/spirituality, risk, and the development of alcohol dependence in female twins. Psychol. Addict. Behav..

[50] Moak ZB, Agrawal A (2010). The association between perceived interpersonal social support and physical and mental health: results from the National Epidemiological Survey on Alcohol and Related Conditions. J. Public Health (Oxf.).

[51] Agrawal A (2017). Differences between White and Black young women in the relationship between religious service attendance and alcohol involvement. Am. J. Addict..

[52] Heath AC (1999). Resiliency factors protecting against teenage alcohol use and smoking: influences of religion, religious involvement and values, and ethnicity in the Missouri Adolescent Female Twin Study. Twin Res..

[53] Zapolski TCB, Pedersen SL, McCarthy DM, Smith GT (2014). Less drinking, yet more problems: understanding African American drinking and related problems. Psychol. Bull..

