# Supplementary material for: Psychosocial moderation of polygenic risk for cannabis involvement: the role of trauma exposure and frequency of religious service attendance
Source: Transl Psychiatry. 2019 Oct 21;9:269. doi: 10.1038/s41398-019-0598-z (PMC6803671; doi:10.1038/s41398-019-0598-z)
Supplement: Supplementary file 1 — Supplementary Table Captions [file 41398_2019_598_MOESM1_ESM.docx]

**Supplementary Table 1.** Moderation of cannabis use and DSM-5 CUD symptom count by trauma exposure and frequency of religious service attendance among EA participants, model 2 results. Model 2 includes the following covariates: age, sex, birth cohort, PCs1-3, PRS*trauma/frequency of service attendance, PRS*age, PRS*sex, PRS*PCs1-3, trauma/frequency of service attendance*age, trauma/frequency of service attendance*sex, trauma*PCs1-3. Boldface indicates estimates with p-value<0.05.

**Supplementary Table 2.** Main effects of Polygenic Risk Scores on cannabis use and DSM-5 cannabis use disorder symptom count in COGA participants of African ancestry

Footnote: Polygenic risk scores are derived from Pasman cannabis initiation GWAS^4^ summary statistics. Covariates include sex, birth cohort, PCs1-3

**Supplemental Table 3.** Traumatic exposures included in the SSAGA-IV
