# Supplementary material for: Psychosocial moderation of polygenic risk for cannabis involvement: the role of trauma exposure and frequency of religious service attendance
Source: Transl Psychiatry. 2019 Oct 21;9:269. doi: 10.1038/s41398-019-0598-z (PMC6803671; doi:10.1038/s41398-019-0598-z)
Supplement: Supplementary file 2 — Supplementary Table 1 [file 41398_2019_598_MOESM2_ESM.docx]

Supplementary Table 1.

|  | Cannabis use ever  (lifetime) | | | DSM-5 Cannabis Use Disorder Symptom Count | | |
| --- | --- | --- | --- | --- | --- | --- |
| *PRS Threshold* | R^2^ | Beta | P-value | R^2^ | Beta | P-value |
| **Trauma Exposure**  **x PRS** |  |  |  |  |  |  |
| p<0.0001 | -- | -- | -- | -- | -- | -- |
| p<0.001 | -- | -- | -- | -- | -- | -- |
| p<0.01 | -- | -- | -- | -- | -- | -- |
| p<0.05 | **0.006** | **0.203** | **0.005** | 0.002 | 0.117 | 0.097 |
| p<0.1 | **0.005** | **0.371** | **0.012** | 0.001 | 0.142 | 0.323 |
| p<0.2 | -- | -- | -- | -- | -- | -- |
| p<0.3 | -- | -- | -- | -- | -- | -- |
| p<0.4 | -- | -- | -- | -- | -- | -- |
| p<0.5 | -- | -- | -- | -- | -- | -- |
| **Frequency of Service Attendance**  **x PRS** |  |  |  |  |  |  |
| p<0.0001 | -- | -- | -- | -- | -- | -- |
| p<0.001 | -- | -- | -- | -- | -- | -- |
| p<0.01 | -- | -- | -- | -- | -- | -- |
| p<0.05 | **0.005** | **0.279** | **0.023** | 0.000 | 0.053 | 0.672 |
| p<0.1 | **0.003** | **0.434** | **0.083** | 0.000 | 0.019 | 0.942 |
| p<0.2 | -- | -- | -- | -- | -- | -- |
| p<0.3 | -- | -- | -- | -- | -- | -- |
| p<0.4 | -- | -- | -- | -- | -- | -- |
| p<0.5 | -- | -- | -- | -- | -- | -- |
| Note: Moderation analyses were only conducted on scores where a statistically significant (p<0.002) main effect was observed. | | | | | | |
