# Supplementary material for: Psychosocial moderation of polygenic risk for cannabis involvement: the role of trauma exposure and frequency of religious service attendance
Source: Transl Psychiatry. 2019 Oct 21;9:269. doi: 10.1038/s41398-019-0598-z (PMC6803671; doi:10.1038/s41398-019-0598-z)
Supplement: Supplementary file 3 — Supplementary Table 2 [file 41398_2019_598_MOESM3_ESM.docx]

Supplementary Table 2.

|  | Cannabis use ever  (lifetime) | | | DSM-5 Cannabis Use Disorder  Symptom Count | | |
| --- | --- | --- | --- | --- | --- | --- |
| Polygenic Risk Score Thresholds (Pasman^8^) | R^2^ | Beta | P-value | R^2^ | Beta | P-value |
| p<0.0001 | 0.000 | 0.014 | 0.693 | 0.001 | 0.029 | 0.401 |
| p<0.001 | 0.000 | 0.003 | 0.933 | 0.000 | 0.011 | 0.746 |
| p<0.01 | 0.001 | 0.036 | 0.300 | 0.003 | -0.058 | 0.118 |
| p<0.1 | 0.000 | 0.001 | 0.982 | 0.001 | -0.042 | 0.226 |
| p<0.2 | 0.000 | 0.016 | 0.644 | 0.001 | -0.033 | 0.340 |
| p<0.3 | 0.000 | 0.022 | 0.552 | 0.001 | -0.033 | 0.356 |
| p<0.4 | 0.000 | 0.024 | 0.515 | 0.001 | -0.042 | 0.249 |
| p<0.5 | 0.000 | 0.029 | 0.441 | 0.001 | -0.043 | 0.243 |
| Footnote: Polygenic risk scores are derived from International Cannabis Consortium cannabis initiation GWAS^4^ summary statistics. Covariates include sex, birth cohort, PCs1-3 | | | | | | |
