# Supplementary material for: Psychosocial moderation of polygenic risk for cannabis involvement: the role of trauma exposure and frequency of religious service attendance
Source: Transl Psychiatry. 2019 Oct 21;9:269. doi: 10.1038/s41398-019-0598-z (PMC6803671; doi:10.1038/s41398-019-0598-z)
Supplement: Supplementary file 4 — Supplementary Table 3 [file 41398_2019_598_MOESM4_ESM.docx]

**Supplemental Table 3.**

| *Assaultive traumatic exposures* |
| --- |
| 1. Have you ever been shot? |
| 1. Have you ever been stabbed? |
| 1. Have you ever been mugged or threatened with a weapon, or experienced a break-in or robbery? |
| *Sexual assaultive traumatic exposures* |
| 1. Have you ever been raped or sexually assaulted by a relative? |
| 1. Have you ever been raped or sexually assaulted by someone not related to you? |
| *Non assaultive traumatic exposures* |
| 1. Have you ever been in a natural disaster like a fire, flood, earthquake, tornado, mudslide or hurricane? |
| 1. Have you ever been held captive, tortured, or kidnapped? |
| 1. Have you ever been diagnosed with a life threatening illness? |
| 1. Have you ever been in a serious accident? 2. Have you ever seen someone being seriously injured or killed? |
| 1. Have you ever unexpectedly discovered a dead body? |
